# EXPLORING THE IMPACT OF RACE-ETHNICITY ON RESPONSE TO WEIGHT-LOSS TREATMENT: RESULTS FROM THE POWR-UP STUDY

**DOI:** 10.1093/geroni/igz038.1833

**Published:** 2019-11-08

**Authors:** Marshall G Miller, Cassandra M Germain, Kathryn N Porter Starr, Martha E Payne, Richard Sloane, Connie W Bales

**Affiliations:** 1 Duke University, Durham, North Carolina, United States; 2 North Carolina A&T State University, Greensboro, North Carolina, United States; 3 Duke University School of Medicine, Durham, North Carolina, United States; 4 Duke University Medical Center, Durham, North Carolina, United States

## Abstract

Racial/ethnic differences in obesity prevalence and in responses to weight-loss treatment between Black and White women are well documented. Whether these differences influence responses to weight-loss treatment among older women is unknown. Therefore, we evaluated racial/ethnic differences among participants in a 6-month weight-loss study with traditional versus higher protein intake. Participants were obese (BMI ≥ 30 kg/m2) community-dwelling women, age 45 years or older, who self-identified as either Black or White. Change in body-weight, 6 minute walk test (6MWT), general health (SF-36), and satisfaction with life (SWL) were evaluated at 0, 4 and 6 months. Both racial groups reduced (ps < 0.01) body weight at 4 and 6 months, with a trend toward more weight loss among White women (p = 0.07), relative to Black women. Other racial/ethnic differences included greater improvements in general health (p = 0.05) and 6MWT (p < 0.05) for White versus Black women at 6 months; these differences persisted after adjusting for treatment group, age/education, and comorbidity. Although racial/ethnic differences in SWL were not observed, significant improvement was observed only among White women (p < 0.01). Interestingly, weight loss was associated with improved 6MWT only among Black women (r = -0.66, p < 0.05) and with general health only among White women (r = -0.44, p < 0.05). Overall, White women experienced greater improvements in health and physical function as a result of weight-loss than did Black women. Further research is needed to identify equitable intervention strategies for the treatment of sarcopenic obesity.

